# Designing a Personalized Digital Patient Support Program for Patients Treated With Growth Hormone: Key Design Considerations

**DOI:** 10.2196/18157

**Published:** 2020-07-29

**Authors:** Sumaira Malik, Clare Moloney, Ekaterina Koledova, Jonathan Reston, John Weinman

**Affiliations:** 1 Atlantis Healthcare London United Kingdom; 2 Endocrinology, Global Medical, Safety and CMO Merck KGaA Darmstadt Germany; 3 Institute of Pharmaceutical Science Kings College London London United Kingdom

**Keywords:** growth hormone, behavior change wheel, somatropin, adherence

## Abstract

**Background:**

Recombinant human growth hormone treatment can optimize growth potential; however, optimal outcomes are not always achieved for several reasons, including poor adherence. The overall objective of this project was to design a patient support program to maximize the chances of treatment success for people being treated with somatropin injection. An approach known as the behavior change wheel was used to enhance the development of the patient support program. The behavior change wheel provided a comprehensive framework to support the design of interventions.

**Objective:**

The aim of this paper was to describe how the steps of the behavior change wheel were applied to the development of a patient support program for individuals with growth hormone deficiency undergoing treatment with somatropin.

**Methods:**

We followed a series of steps that align to tenets of the behavior change wheel, namely, a narrative literature review to identify which behaviors needed to change and the potential drivers of and barriers to the behaviors, the selection of an intervention strategy and discrete behavior change techniques, and, finally, intervention specification.

**Results:**

A recent systematic review identified a range of potentially modifiable factors found to have an influence on patient adherence to growth hormone treatment. Insights from the systematic review were used to guide the development of a patient support program. The final design of the patient support program consisted of four elements: (1) a personalization questionnaire to tailor support for each individual, (2) tailored reminder and support SMS text messages, (3) nurse-led phone calls, and (4) Easypod connect, an automated electronic autoinjector drug-delivery device with a transmitter and connection platform for Saizen (somatropin) that allows automatic recording, storage, and transmission of drug-usage data, thus providing insight into suboptimal adherence.

**Conclusions:**

The patient support program that was designed is currently being piloted with patients to assess engagement with the program and determine its impact on patient outcomes. Results from the pilot will be used to further refine the program to ensure it meets user needs.

## Introduction

Recombinant human growth hormone (r-hGH; somatropin) treatment is well established for children and adolescents with growth hormone deficiency [1]. The goal of this treatment is to optimize growth potential, so that the expected target height (based on a normal growth curve for each individual) is achieved by adulthood [2]. Evidence has demonstrated the efficacy of growth hormone treatments to improve growth in children with growth hormone deficiency compared to that of children whose growth hormone deficiency is not treated [3].

The impact of growth hormone deficiency goes beyond reduced growth potential. Research has shown that children with growth hormone deficiency may also experience a psychosocial burden as a result of being physically different from their peers [3,4]. This burden can continue into adulthood, particularly if short stature persists, limiting both personal and professional success [3] as well as potentially having an impact on long-term health, since, as adults, they may also have an increased risk of cardiovascular diseases [5]. With prevalence estimates for growth hormone deficiency from 1.8 to 2.9 per 10,000 in Europe and the United States [6-8], the potential of treatment success to benefit the health economy as well as patients and their families is significant [3].

**Figure 1:**
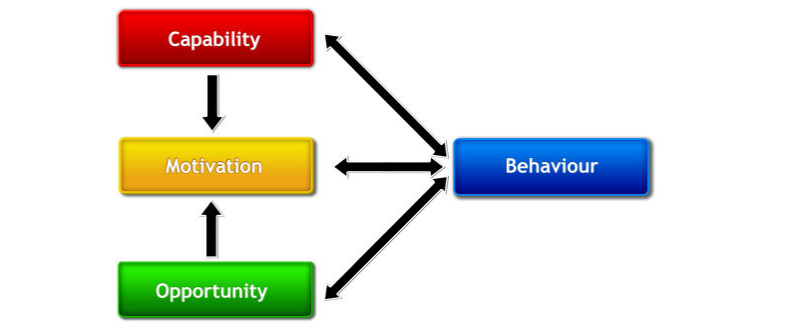
Capability, opportunity, motivation, and behavior framework.

The overall objective of this project was to design a patient support program to maximize the chances of treatment success for people treated with somatropin by injection. An approach known as the behavior change wheel [9] was used to enhance the development of the patient support program. The behavior change wheel provided a comprehensive framework to support the design of the interventions allowing designers to move from behavior analysis to intervention design using evidence-based techniques and behavior change theory. Central to the behavior change wheel is a framework called the capability, opportunity, motivation, and behavior model (COM-B) [9] (Figure 1). The COM-B model proposes that a person’s health behaviors are driven by a range of factors, which can be grouped under three broad headings: *capability*, their physical or psychological ability to engage in the behavior, for example, in the context of adherence to medication, factors such as reduced cognitive functioning, mobility problems, or poor treatment comprehension may affect an individual’s ability to understand how to physically administer their treatment; *motivation*, the internal thoughts and emotions that influence individual decision making in relation to the behavior, for example, beliefs about the need for treatment or the seriousness of the illness may affect how motivated an individual is to try a new treatment or depression as a result of their health condition can reduce their motivation to adhere to a treatment regimen; and *opportunity*, the external factors which make it possible to engage in the behavior, for example, having a supportive social network and easy access to healthcare resources. In addition to each component influencing medication-related behavior directly, opportunity and capability may affect motivation, and thus also influence behavior in this manner. Jackson and colleagues [10] successfully applied this model to treatment adherence behaviors. While there are similarities in factors for adherence across a patient population, it is important to note that each patient has their own unique needs and set of beliefs that have an impact on their ability to adhere to treatment [11]. Offering personally tailored self-management or adherence interventions to suit their individual needs is the most effective way to ensure patients understand and adopt targeted behaviors, including adherence to r-hGH treatment [11-13]. Tailored interventions and support have also been shown to be more cost-effective [14], an important consideration in the sustainability of support services.

Another crucial component of the behavior change wheel is the selection of appropriate techniques to change behavior. In this paper, we describe how the steps of the behavior change wheel were applied to the development of a patient support program for individuals with growth hormone deficiency who were prescribed somatropin; the duration of individual treatments varies depending on age at time of diagnosis (usually early childhood), and in order to reach full height potential, are continued up to the age when bone growth stops (as the individual reaches adult height). The patient support program was designed to incorporate a large digital component, while also maintaining a strong human support element to personalize and complement the digital aspects of the intervention. Digital interventions that incorporate a wide range of communication channels such as SMS text messaging, interactive websites, apps, and electronic devices are thought to have great potential for this patient population, given the popularity of digital media among young people [15]; however, despite this, maintaining engagement with digital interventions can be difficult with many studies reporting high levels of dropouts or nonusage [16]. Personalized messaging and multicomponent interventions including both digital and nondigital aspects have been shown to be more effective for behavior change than nontailored messaging and single-component interventions [17]. Yardley et al [16] proposed several general principles and considerations for maximizing engagement with digital behavior-change interventions; they stressed that, most importantly, the intervention should be relevant to the user, ie, it should address an unmet need and should be tailored to the specific situation, needs, and motivations of the user [16]. Utilizing the behavior change wheel and the COM-B framework allowed us to identify the drivers that underlie treatment-related behavior in growth hormone deficiency patients and caregivers, and thus, to create a program with relevant tailored content. Further suggestions [16] included adding a human element to help maintain motivation and ongoing adherence to the digital components of the intervention, ensuring the intervention content was accessible and engaging for people with different levels of health literacy, and taking an iterative approach to intervention development and evaluation to refine the intervention to ensure it would continue to meet user needs. These considerations were also used to guide the design of the patient support program.

## Methods

### Overview

**Figure 2:**
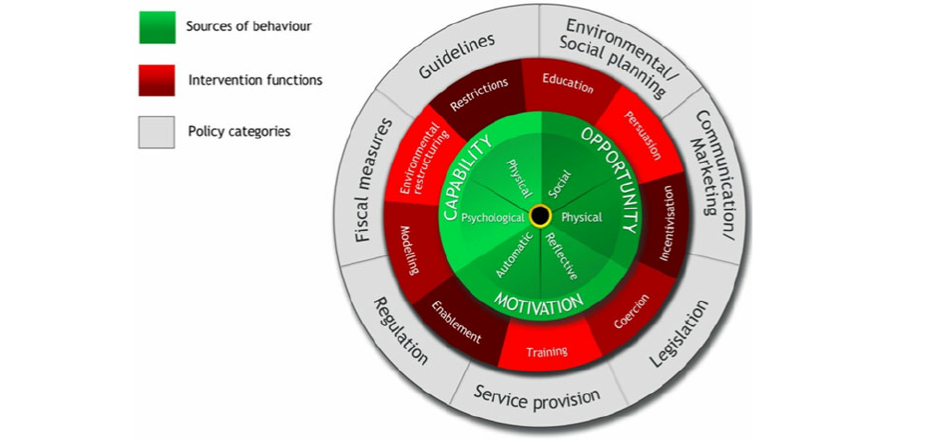
Behavior change wheel.

We followed a series of steps that were aligned to tenets of the behavior change wheel [9], namely, identifying behaviors that needed to change, identifying the potential drivers of and barriers to the behavior, selecting an intervention strategy and discreet behavior change techniques, and specifying an intervention (Figure 2).

### Step 1

A narrative review of the literature was carried out to understand problems associated with treatment management in growth hormone deficiency and to select and specify the target behavior. Literature searches were conducted using CINAHL, Medline, PsychINFO (via EBSCOHost), and the Cochrane Register for Controlled Trials and Systematic Reviews. The following keywords were used: “growth hormone,” “human growth hormone” (MeSH), “child” (MeSH), “adolescent” (MeSH), “patient compliance” (MeSH). Literature was limited to papers written in English and no date restrictions were applied. The reference lists and citations of publications that were identified were also searched for additional papers on the topic.

### Step 2

Underlying barriers and drivers were defined using insights from a 2018 systematic review [18] looking at the range of potentially modifiable factors that have been found to influence levels of adherence to growth hormone treatment; these factors were grouped according to the COM-B framework. The findings from this review provided direction for selecting the types of interventions that would be needed to effectively target these factors in digital interventions.

### Step 3

Intervention options were identified using the behavior change wheel, which proposes nine intervention functions: education, persuasion, incentivization, coercion, training, restriction, environmental restructuring, modeling, and enablement. We used APEASE criteria (acceptability, practicability, effectiveness or cost-effectiveness, affordability, safety or side effects, and equity) [19] to guide our selection of the most relevant intervention functions. We also engaged a team of specialists in health psychology with expertise in behavior change.

### Step 4

Based on the factors identified by Graham et al [18] and the intervention functions that were selected, a list of relevant behavior change techniques was defined to address each COM-B factor.

### Step 5

The full intervention was mapped out, which involved selecting delivery channels (SMS text messages, nurse-led calls, Growlink app), applying the appropriate behavior change techniques to develop content, and designing a personalization questionnaire to tailor the patient experience. In addition, a measurement strategy was designed to evaluate the effectiveness of the intervention.

## Results

### Step 1: Defining the Problem

A narrative review of the literature on growth hormone deficiency treatments found that despite the potential of r-hGH treatment to optimize growth potential, optimal outcomes were not always achieved; it was proposed that poor adherence was a key contributor to this [2,20,21]. The levels of nonadherence that were reported varied, particularly since definitions and assessment methods differed between studies, but with the proportion of nonadherent patients ranging from 5% to 82% across several indications for which growth hormone therapy is used [2] and with 7% to 71% of patients with growth hormone deficiency found to be nonadherent to treatment [18], it was clear that there was room to improve adherence in this population [2]. Adherence to somatropin injections was selected as a target behavior and outcome for the design of the patient support program. Table 1 further describes the target behaviors.

**Table 1:** Target behavior description.

Target behavior	Adherence to somatropin injection
Who needs to perform the behavior?	Pediatric patients with growth hormone deficiency or their caregiver
Where will they do it?	At home
How often will they do it?	This will vary depending on individual needs and will be determined by the prescription issued by their health care provider

### Step 2: Behavior Diagnosis

In work relevant to this study, Graham et al [18] conducted a systematic review of pediatric nonadherence to r-hGH treatment using the COM-B framework to identify potentially modifiable factors. Key factors were identified in the review: within the scope of capability—knowledge and understanding of the condition, a lack of understanding of the consequences of missed r-hGH doses, forgetting to administer the medication, or poor administration technique; within the scope of opportunity—inadequate contact with health care providers and the quality of the health care provider–patient relationship, as well as the discomfort and pain associated with daily injections; and within the scope of motivation—the long duration of treatment and dissatisfaction with growth response results.

These findings were largely reflective of the insights gained from our narrative review of growth hormone and adherence literature; they suggested a need to address a range of factors related to patients receiving growth hormone therapy, their caregivers, and growth hormone deficiency and its treatment to ensure optimal adherence.

### Steps 3 and 4: Implementation Strategy and Behavior Change Technique Selection

While all nine of the interventions listed in the behavior change wheel were found to be relevant to nonadherence to treatment in growth hormone deficiency, we selected the five intervention functions that we felt were the most relevant and the most appropriate to address within the patient support program; these were education, persuasion, training, modeling, and enablement. This decision was based on a combination of practical considerations such as affordability and cost-effectiveness as well as on behavior change expertise from a team of specialists in health psychology. Table 2 shows the relationship between the factors identified in the COM-B, intervention functions, and selected behavior change techniques. An expanded version of Table 2 including COM-B categories, intervention functions, and intervention content is available in Multimedia Appendix 1.

**Table 2:** COM-B adherence factors and behavior change techniques.

Adherence factors	Behavior change techniques
Long duration of treatment	Framing or reframing
Dissatisfaction with treatment outcome	Self-monitoring Framing or reframing Reduce negative emotion
Knowledge and understanding of condition	Credible source Information on health consequences
Discomfort or pain from daily injection	Instruction of how to perform the behavior Problem solving Verbal persuasion of capability Reduce negative emotion
Lack of understanding of the consequences of missed doses	Information on health consequence Salience of consequences
Forgetting	Feedback on behavior Self-monitorin Prompts or cues Problem solving
Health care provider–patient communication	Demonstration of the behavior Problem solving Verbal persuasion of capability
Poor injection technique	Instruction on how to perform the behavior Demonstration of the behavior

### Step 5: Mapping the Design of the Patient Support Program

#### Overview

Based on these findings, as well as on insights from digital behavior change intervention design principles and behavior change theory, a multicomponent digital patient support program was designed for patients receiving treatment with somatropin injection (see Table 2 for example content). The patient support program consists of four components: personalization screening, SMS text messages, nurse-led phone calls, and an eHealth component.

#### Personalization Screening Questionnaire

A set of personalization questions was created based on the drivers of adherence that were identified in step 2. The design includes different questions for caregivers and patients, allowing for support to be tailored to both or either, depending on who is taking part in the patient support program. Patients and caregivers will be asked to complete the screening questionnaire when they join the patient support program and will be rescreened every 12 weeks. This is to ensure that support can be flexible to changing needs and to allow for review of intervention effectiveness by monitoring changes to beliefs and to support needs. The personalization screening questionnaire will be used to determine topic priority and focus of the SMS text messages and of the nurse-led components of the program.

#### SMS Text Messages

These include medication reminders as well as tailored intervention messages to address adherence factors that have been identified as important through the personalization screening questionnaire.

#### Nurse-Led Phone Calls

Patient support program nurses will be trained to deliver brief, evidence-based interventions to address the adherence barriers that were identified. The content and order of these telephone calls will be tailored to the needs of each individual patient or caregiver based on personalization screening questionnaire responses.

#### eHealth Component

The Easypod is an automated electronic autoinjector drug-delivery device with a transmitter and web-based connection platform for Saizen (somatropin) that automatically inserts a needle and delivers a preset dose to the patient. The device allows automatic recording, storage, and transmission of drug adherence data, thus providing insight into suboptimal adherence (through dose frequency) and its resulting effect on growth. Patients and caregivers can access the information via a patient app (Growlink), and physicians and nurses can access the information via the web-based Easypod connect platform.

## Discussion

### Principal Findings

The purpose of this paper was to describe the development of a patient support program aimed at supporting patients who have been prescribed Saizen in order to maximize their growth potential. A structured approach based on the behavior change wheel and the COM-B framework was used to identify the drivers of behavior and to systematically explore how these could be addressed through a pharma-funded patient support program.

The final design of the patient support program incorporated a patient-centric digital component featuring an online app linked to the injection device and frequent SMS text message delivery. The digital platform offers a convenient method for patients, caregivers, and health care providers to interact, to share, and to review adherence data for each patient. The addition of SMS text messaging allows adherence reminder messages and intervention content that was designed to address nonadherence to be sent to individual patients. SMS text messages have been successfully used in behavior change interventions as both reminders for appointments or for treatments [22], as well as to deliver interventions to change patient beliefs and to improve adherence [2]. A 2011 meta-analysis [23] of the efficacy of SMS text message reminders showed that they were effective across all age groups; there were also no differences in effect based on the timing of the messages or the rate that the messages were sent [23]. Messaging interventions have been shown to improve adherence to medications across a wide variety of clinical applications including asthma, antiretroviral treatments, and schizophrenia, in both adult and pediatric populations [22,24,25]. The effect of personalized messaging on behavior has also been shown to be more effective than that of nontailored messaging [17]. In line with the recommendations of Yardley et al [16], tailored content may also help increase engagement with digital interventions. In this patient support program, tailored content was achieved through a personalization screening questionnaire designed to determine the content and sequence of SMS text messages and nurse-led phone calls for delivery of the intervention; the online app was also designed to provide personalized adherence feedback.

Personalized telephone calls with a specialist nurse add a human element to the patient support program which may help to support continued engagement. Evidence has shown that nurses can successfully be trained to use behavior change techniques and to implement motivational interviewing techniques by a simple, brief course [26,27]. Nurse-led calls that implement motivational interviewing principles and that teach behavior change techniques have been shown to result in meaningful behavior change [28,29]. In addition, telephone-based support has been shown to be an effective delivery channel for promoting behavior change across different health conditions such as smoking cessation, increasing physical activity, and improving diet [30], as well as demonstrating a positive impact on treatment adherence [31,32]. Research has also shown that, in general, multicomponent interventions are more successful in having an impact on behavior than single-component interventions are [33]. Thus, the nurse-led coaching phone calls are likely to enhance and complement the support provided through the digital components.

Another important consideration for any patient support program is the level of health literacy. Yardley et al [16] argued that digital behavior change interventions should be accessible and engaging for people with low levels of health literacy and should also be acceptable and usable for those with higher levels of health literacy. All content that was developed for the patient support program was reviewed by an experienced health psychologist and writer, and by a creative team to ensure the pitch, tone, and level was appropriate for the range of health literacy levels in the patient group.

### Future Work

Our patient support program will be piloted with the aim of establishing its perceived acceptability and usefulness. The evaluation of the pilot patient support program will be conducted using an observational (real-world evidence) within-subjects design. Three categories of data will be captured: operational data, user experience data, and impact data. The primary outcome will be patient perception of the helpfulness of the patient support program. Secondary outcomes will include change in adherence and change in quality of life over the course of the patient support program, and multiple regression modeled predictors of these outcomes. Finally, data will be captured to identify ways in which various components of the patient support program could be improved, in line with the principles of continuous development in patient support program design, behavior change technique, and digital interventions proposed by Michie et al [34]. These data will be captured during routine operation via the patient support program (such as number and length of sessions), supplementary behavior-changing customer relationship management, and follow-up evaluation surveys.

### Strengths and Limitations

The patient support program was developed using a systematic and structured approach, by drawing on relevant literature, and using appropriate evidence-based behavior change theories and frameworks. To the best of our knowledge, this represents a novel approach to the development of patient support programs in growth hormone deficiency; however, despite this, the use of the behavior change wheel inevitably required that the intervention development team make subjective and practical decisions regarding the most appropriate strategies and delivery channels. Since there was limited data on the most appropriate intervention channels and implementation of behavior change techniques for this patient population; the team drew from their own experience working in this patient population to guide some of these decisions.

The first part of the design process involved a narrative review of relevant literature. While this review captured a large volume of relevant literature, the methods were not exhaustive. Key search terms were provided, but to ensure that all relevant papers had been captured in an entirely reproducible format, a full systematic review was required. To be efficient, this paper focused, where possible, on meta-analyses and systematic reviews such as [18] which partially summarized large quantities of the literature.

### Conclusions

Using the approach outlined by the behavior change wheel, a multicomponent patient support program for patients with growth hormone deficiency (and their caregivers) to achieve their growth potential through adherence using the Easypod connect platform was developed. By incorporating personalization screening questionnaires into the patient support program, personalized intervention messages and nurse-led phone support can be offered to patients. Work is underway to implement and validate the patient support program, to establish whether it can improve adherence in a real-world setting.

## Appendix

Multimedia Appendix 1 Expanded Table 2.

## Abbreviations

COM-B: capability, opportunity, motivation, and behavior model
MeSH: medical subject heading (index term)
r-hGH: recombinant human growth hormone
