# Supplementary material for: Designing a Personalized Digital Patient Support Program for Patients Treated With Growth Hormone: Key Design Considerations
Source: J Med Internet Res. 2020 Jul 29;22(7):e18157. doi: 10.2196/18157 (PMC7424476; doi:10.2196/18157)
Supplement: Multimedia Appendix 1 [file jmir_v22i7e18157_app1.doc]

Expanded Table 2

| **Adherence factors** | **COM-B Category** | **Intervention Function** | **Behavior Change Techniques (BCTs)** | **Description of intervention strategy** | **Example content** |
| --- | --- | --- | --- | --- | --- |
| Long duration of treatment | Motivation | Persuasion  Enablement | Framing/reframing | Nurse coaching calls to encourage individual to adopt different ways of thinking in order to change thoughts or feelings about length of treatment.  Personalised SMS | Example SMS: *Using Saizen over a long period of time can lead to excellent catch-up growth. So, carry on taking it as your doctor has prescribed*. |
| Dissatisfaction with treatment outcome | Motivation | Persuasion Education | Self-monitoring  Framing/reframing  Reduce negative emotion | Feedback loop and adherence monitoring through the grow link app  Personalised SMS  Nurse coaching calls to reduce negative emotions around the success of treatment and suggest the deliberate adoption of a new perspective on treatment and its purpose e.g. treatment may help a patients emotional wellbeing, rather than just increasing height. | Example Nurse Call Activity: Thought balancing exercise to recognise and realign unhelpful thoughts. |
| Knowledge/ understanding of condition | Capability (Psychological) | Education | Credible source  Information on health consequences | Personalized SMS  Nurse coaching calls to explore knowledge gaps. | Provision of factual information from a nurse advisor to tackle common misunderstandings/ knowledge gaps.  Particular emphasis on explaining the role of treatment in managing the condition and the consequences of non-adherence, such as less ‘catch-up’ growth. |
| Discomfort/pain from daily injection | Opportunity (Physical) | Training  Education  Enablement  Persuasion | Instruction of how to perform the behavior  Problem-solving  Verbal persuasion of capability  Reduce negative emotion | Training videos  Nurse coaching calls to support patients with self-injection problems  SMS messaging to promote self-efficacy and provide encouragement | Example SMS: *Don’t forget your Saizen. If injections are painful, a distraction (even focusing on the wall) and massaging the area afterwards may help.* |
| Lack of understanding of consequences of missed doses | Capability (Psychological) | Education | Information on health consequences  Salience of consequences | Personalized SMS  Nurse coaching calls to explore knowledge gaps and misconceptions | Example SMS: *Just two missed injections per week could lead to less growth each year than someone who has all their injections*. |
| Forgetting | Capability (Psychological) | Enablement  Persuasion  Modeling | Feedback on behavior  Self-monitoring  Prompts/cues  Problem-solving | Growlink app feedback loop and opportunity to self-monitor adherence  Nurse coaching calls to encourage patients to identify their own strategies for remembering the treatment  Reminder SMS | Example Nurse Call Activity: Problem-solving exercise to prompt the person to analyze factors that might be affecting behaviors such as remembering treatment, administering treatment, and helping them to generate strategies to overcome such barriers. |
| HCP-Patient communication | Opportunity (Social) | Enablement  Persuasion | Demonstration of the behavior  Problem-solving  Verbal persuasion of capability | Personalized SMS  Nurse coaching calls to improve skills for getting the most out of HCP consultations and boost confidence in patient’s ability to communicate their needs effectively. | Example Nurse Call Activity: Exploring strategies to boost confidence in speaking with HCPs about individual needs and concerns  Setting clear plans and goals in relation to the strategies they are going to implement to boost confidence in next HCP consultation. |
| Poor injection technique | Capability  (Physical) | Modeling  Training | Instruction on how to perform the behavior  Demonstration of the behavior | Training videos  Support through nurse coaching calls.  Personalized SMS | Example SMS: *Don’t forget to always clean your skin with antibacterial soap and water before injecting.* |
